# A Miniature Magnetic-Force-Based Three-Axis AC Magnetic Sensor with Piezoelectric/Vibrational Energy-Harvesting Functions

**DOI:** 10.3390/s17020308

**Published:** 2017-02-08

**Authors:** Chiao-Fang Hung, Po-Chen Yeh, Tien-Kan Chung

**Affiliations:** 1 Department of Mechanical Engineering, National Chiao Tung University, Hsinchu 30010, Taiwan; ithildin.me01g@nctu.edu.tw (C.-F.H.); scarletleaf.me02g@nctu.edu.tw (P.-C.Y.); 2 International College of Semiconductor Technology, National Chiao Tung University, Hsinchu 30010, Taiwan

**Keywords:** AC magnetic sensor, magnetic force, piezoelectric, mechanical, 3-axis, energy harvesting

## Abstract

In this paper, we demonstrate a miniature magnetic-force-based, three-axis, AC magnetic sensor with piezoelectric/vibrational energy-harvesting functions. For magnetic sensing, the sensor employs a magnetic–mechanical–piezoelectric configuration (which uses magnetic force and torque, a compact, single, mechanical mechanism, and the piezoelectric effect) to convert *x*-axis and *y*-axis in-plane and *z*-axis magnetic fields into piezoelectric voltage outputs. Under the *x*-axis magnetic field (sine-wave, 100 Hz, 0.2–3.2 gauss) and the *z*-axis magnetic field (sine-wave, 142 Hz, 0.2–3.2 gauss), the voltage output with the sensitivity of the sensor are 1.13–26.15 mV with 8.79 mV/gauss and 1.31–8.92 mV with 2.63 mV/gauss, respectively. In addition, through this configuration, the sensor can harness ambient vibrational energy, i.e., possessing piezoelectric/vibrational energy-harvesting functions. Under *x*-axis vibration (sine-wave, 100 Hz, 3.5 g) and *z*-axis vibration (sine-wave, 142 Hz, 3.8 g), the root-mean-square voltage output with power output of the sensor is 439 mV with 0.333 μW and 138 mV with 0.051 μW, respectively. These results show that the sensor, using this configuration, successfully achieves three-axis magnetic field sensing and three-axis vibration energy-harvesting. Due to these features, the three-axis AC magnetic sensor could be an important design reference in order to develop future three-axis AC magnetic sensors, which possess energy-harvesting functions, for practical industrial applications, such as intelligent vehicle/traffic monitoring, processes monitoring, security systems, and so on.

## 1. Introduction

To date, magnetic sensing technology is still one of the important research fields with respect to sensors, and has created many energy, commercial–electronic, medical, and industrial applications. Novel magnetic sensors, using smart materials, are used for a number of applications, including for condition monitoring of current-carrying wires in smart grid applications [1,2,3], magnetic-field detection of magnetic read–write heads in computer hard disks and other data-storage devices [4], magnetic-flux detection of interlocking nails in bone-fracture surgeries [5,6], and magnetic-flux detection/conversion of magnetic energy-harvest-powered wireless sensing systems [7], amongst others. Recently, magnetic sensors have developed rapidly in practical industrial applications, which require a sensing range of a few gauss (such as for application in vehicle/traffic monitoring, processes monitoring, security systems, and so on) [8]. Based on the magnetic-sensing principles for these few-gauss-range applications, magnetic sensors are divided into several representative categories. In these categories, currently, the most developed and comprehensively-used are Hall Effect magnetic sensors [9] and anisotropic magnetoresistive (AMR) magnetic sensors [10,11]. However, compared to Hall Effect and AMR magnetic sensors, microelectromechanical systems (MEMS) resonant magnetic sensors with movable structures have some advantages, such as low-costs, larger output signals, and high sensitivities [12].

Regarding MEMS magnetic sensors, owing to their movable structures, recently, researchers have demonstrated Lorentz-force-based MEMS magnetic sensors [12]. However, these sensors have to be powered/enabled by applying currents. Furthermore, in order to enhance the effect of the Lorentz force, the current-carrying wires of the sensors must be long and thin, which increase resistance, consequently produce more heat dissipation, and eventually increase power consumption. On the other hand, some researchers have demonstrated magnetoelectric-based MEMS magnetic sensors [13,14,15,16,17], which also use moveable structures [17]. However, an additional DC magnetic field is typically needed to enable the magnetic sensing functions of the sensor. Providing this field requires additional energy supply and, thereby, increase the overall power consumption with respect to the system. Therefore, magnetoelectric-based MEMS magnetic sensors, as well as the Lorentz-force-based MEMS magnetic sensors, have power consumption issues. In addition, according to the developing trend of smart sensing technology, the above-mentioned applications can further integrate with a wireless transmission module in order to become a wireless sensor module. However, sometimes the wireless sensor module is located in hard-to-reach areas [18] and, thus, suffer power supply problems [19]. Thus, we believe that if these sensors can have energy-harvesting functions, the sensors not only can be self-powered, but can also power other devices, such as wireless transmission modules (i.e., achieving a self-powered wireless sensor module). In order to add energy-harvesting functions to sensors, combining magnetic sensors with piezoelectric materials (rather than using magnetoelectric-based approaches) would be one of best solutions.

To date, several representative piezoelectric-based magnetic sensors and magnetic energy harvesters have been demonstrated in the literature [3,20,21,22,23,24]. Both, the sensing and energy harvesting approaches, use magnet-induced magnetic force interactions to deflect/deform a single piezoelectric cantilever beam in order to produce piezoelectric voltage responses. However, the single-beam design can only be used to sense a single-axis magnetic field. On the other hand, researchers have recently demonstrated piezoelectric-based, single-mechanical-mechanism three-axis accelerometers and energy harvesters [25,26,27]. That is, by using compact single-mechanical mechanisms (for example, a cross-linked beam), the accelerometers and the energy harvesters can achieve three-axis mechanical motion-sensing and three-axis vibrational energy-harvesting, respectively. According to this, by combining a magnetic material and a piezoelectric-based, single-mechanical mechanism, we can achieve a novel single-mechanical-mechanism-configured, magnetic-force-based, three-axis AC magnetic sensor with piezoelectric/vibrational energy-harvesting functions. Our proposed sensor has the same advantages (i.e., being cost-effective and space-efficient) as commercial three-axis magnetic sensors [28]. Moreover, regarding our proposed sensor with respect to specific applications, some researchers have recently reported a three-axis current sensor for evaluating the integrity of concentric neutrals in underground power distribution cables [29], and an AC magnetic field sensing system for a motion tracking applications [30]. We think that these two applications can more clearly show the practical applications of our sensor (for more details of these applications, please see the Supplemental Materials). Thus, in this paper, we report a three-axis AC magnetic sensor with piezoelectric/vibrational energy-harvesting functions. The most important features of the sensor are the compact, single-mechanical-mechanism-configured, three-axis magnetic field sensing and the three-axis vibrational-energy harvesting. The design, fabrication, testing, and results of the sensor are described in the following sections.

## 2. Design

**Figure 1:**
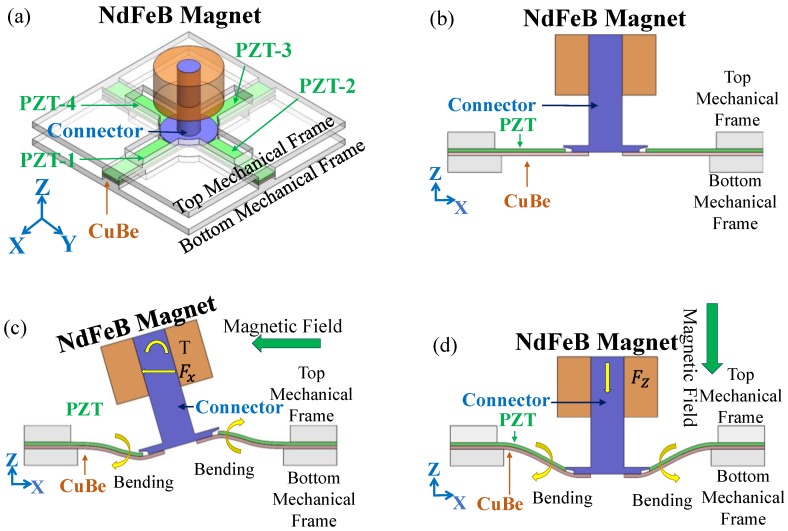
Magnetic-sensing principle of the 3-axis magnetic-force-based AC magnetic sensor: (**a**) isometric view of the sensor. The sensor is under (**b**) initial/static state; zero magnetic field and zero ambient vibration, (**c**) *x*-axis magnetic field, and (**d**) *z*-axis magnetic field.

The design of the sensor is shown in Figure 1a. The sensor is comprised of four piezoelectric lead-zirconate-titanate/copper-beryllium (PZT/CuBe)-layered cantilever beams, a neodymium iron boron (NdFeB) magnet, a connector, and top and bottom mechanical frames. The free end of the CuBe sheet of each PZT/CuBe beam is connected by the connector. The fixed end of each PZT/CuBe beam is clamped by the top and bottom mechanical frames. The four beams are connected as a cross-linked-beam. Finally, the NdFeB magnet is fixed on the surface, at the center of the cross-linked-beam. The magnetic sensing principle of the sensor is shown in Figure 1b,d. In Figure 1b,d, the sensor is under an initial/static state (i.e., zero magnetic field and zero ambient vibrations), an *x*-axis (in-plane) magnetic field, and a *z*-axis (out-of-plane) magnetic field, respectively. When the *x*-axial (or *y*-axial) magnetic field is applied to the sensor, as shown in Figure 1c, the NdFeB magnet (magnetization direction is along the *z*-axis) experiences a magnetic force (Fx (or Fy)) and torque (T). The magnetic force and torque rotate the NdFeB magnet and, therefore, deform the two *x*-axis (or *y*-axis) cantilever beams. Because of the piezoelectric effect, the deformed beams produce voltage outputs. Thus, the sensor achieves *x*-axis (or *y*-axis) magnetic-field sensing. When the *z*-axial magnetic field is applied to the sensor, as shown in Figure 1d, the NdFeB magnet is subjected to a magnetic force (Fz) and, thus, is projected along the *z*-axis; the *z*-axial projection deflects the beams. Due to the piezoelectric effect, the deformed beams produce voltage outputs. Therefore, the sensor achieves *z*-axis magnetic-field sensing.

In addition, when the sensor does not need to sense magnetic fields (i.e., magnetic sensing function is disable or in standby mode), the sensor can be used as a three-axis piezoelectric/vibrational energy harvester, which converts three-axis ambient vibration into voltage outputs (i.e., the vibrational energy harvesting function is enabled or in active mode). The piezoelectric/vibrational energy-harvesting principle of the sensor is fundamentally the same as that of the three-axis energy harvester, previously report by our group [27]. Thus, the details of the principles are not described in this work.

## 3. Fabrication

**Figure 2:**
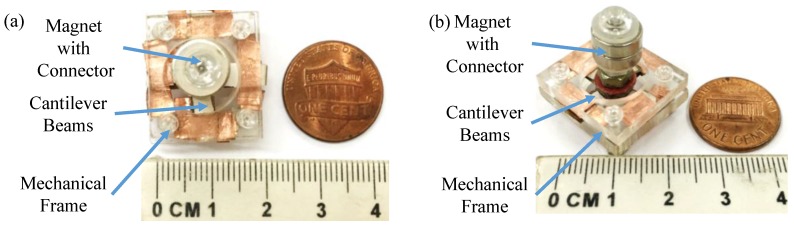
The photographs of the fabricated AC magnetic sensor: (**a**) top view and (**b**) isotropic view.

The fabrication of the sensor comprises three parts: (I) mechanical frames; (II) PZT/CuBe layered cantilever beams; and (III) a connector with a NdFeB magnet. First, acrylic plates were machined to serve as the top and bottom mechanical frames. Second, a PZT-5H sheet (length × width × thickness: 7.5 mm × 4 mm × 1 mm) was fixed on a CuBe sheet (length × width × thickness: 9 mm × 4 mm × 0.25 mm) as a PZT/CuBe-layered cantilever beam. Third, the screw, shim, and NdFeB magnet were assembled to serve as a connector with a NdFeB magnet. Finally, the fixed end of each PZT/CuBe beam was clamped by the top and bottom mechanism frames, while the free end of each beam was connected by the connector with the NdFeB magnet. The fabricated sensor is shown in Figure 2. The dimensions of the sensor and its components are summarized in Table 1.

## 4. Testing

### 4.1. Magnetic Sensing Functions

Before quantitatively testing the performance of the AC magnetic sensor, we have to qualitatively confirm that the deflection, bending, and torsion of the cross-linked-beams of the sensor, under different magnetic fields, are in line with our design (i.e., design validation). To do this, we used a high-speed camera with a controlling computer [27] in order to record the motion of the sensor under the *x*-axis magnetic field, which is produced using a standard commercial electromagnet with a function generator. From the motion recording, we were able to observe and analyze the beams’ corresponding deflections, bending, and torsion.

**Figure 3:**
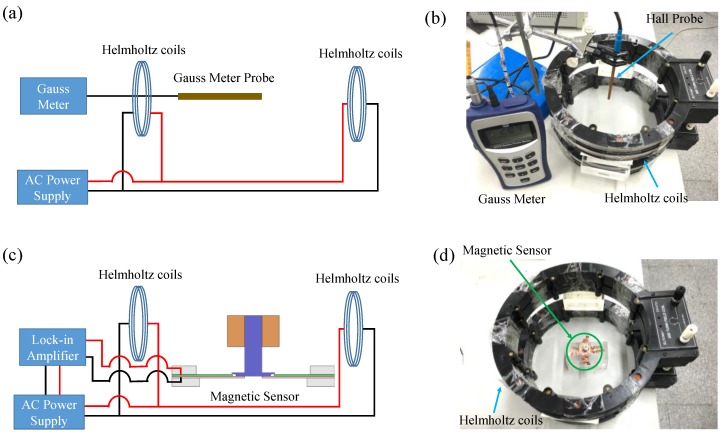
Testing setup of the quantitative test. (**a**) Illustration and (b) photograph of the Hall probe placed in the central area of the coils. (**c**) Illustration and (**d**) photograph of the AC magnetic sensor placed in the central area of the coils.

After qualitative test, a quantitative test is conducted. An illustration and photograph of the quantitative test of the sensor are shown in Figure 3. The testing process is described as follows: First, according to the specification of the Helmholtz coils, we apply an AC voltage to the Helmholtz coils in order to produce an AC magnetic field. Consequently, we placed the Hall probe of a gauss meter to the central location of the coils in order to measure the magnitude of the AC magnetic field, as shown in Figure 3a,b. After this, we tuned the frequency and magnitude of the applied AC voltage to ensure that the magnitude of the produced AC magnetic field remained the same at different frequencies; that is, providing a constant AC magnetic field at different frequencies to the central part of the coils. After using the Hall probe of the gauss meter to measure the magnitude of the magnetic field produced by the coils, we removed the Hall probe and the gauss meter, and, consequently, used a position aligner in order to place the magnetic sensor in the same central location of the coils for further testing, as shown in Figure 3c,d. Under the AC magnetic field, the sensor produced a corresponding piezoelectric voltage output. Therefore, by setting the reference signal as the AC voltage produced by the AC power supply, we used a lock-in amplifier in order to measure piezoelectric voltage output. Based on the testing setup, the resonant frequency of the sensor was measured using a frequency sweeping approach. During frequency sweeping, the gauss meter was used to ensure that the magnitude of the magnetic field remained the same when the frequency of the magnetic field was varied. By comparing the frequencies of the AC magnetic field and the voltage output of the sensor, the resonant frequency of the sensor could be determined. After obtaining the resonant frequency of the sensor, sensitivity measurements of the sensor were conducted. To measure the sensitivity of the sensor, we recorded the voltage outputs of the sensor when it was operated in the resonant mode, but with different magnetic field magnitudes. According to the correlation between the recorded voltage response and the magnetic field magnitudes, the sensitivity of the sensor could be estimated.

### 4.2. Piezoelectric/Vibrational-Energy-Harvesting Functions

**Figure 4:**
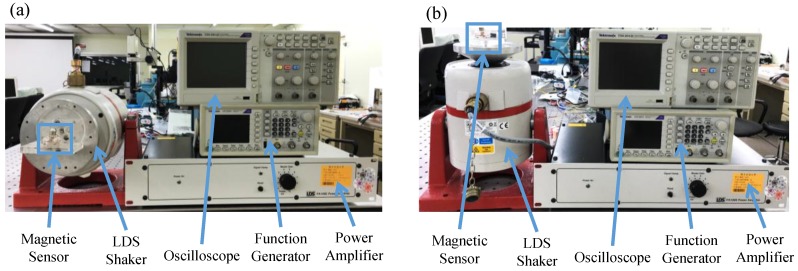
Testing setup of the piezoelectric/vibrational energy-harvesting functions of the sensor under (**a**) *x*-axis vibration and (**b**) *z*-axis vibration.

A photograph of the piezoelectric/vibrational energy-harvesting test [27] is shown in Figure 4. A function generator and a power amplifier were used to operate a Ling Dynamic Systems (LDS) shaker with different orientations in order to generate *x*-axis and *z*-axis vibrations in the sensor, respectively, as shown in Figure 4a,b. An oscilloscope was used to record the voltage outputs of the sensor.

## 5. Results and Discussion

### 5.1. Magnetic-Sensing Functions

**Figure 5:**
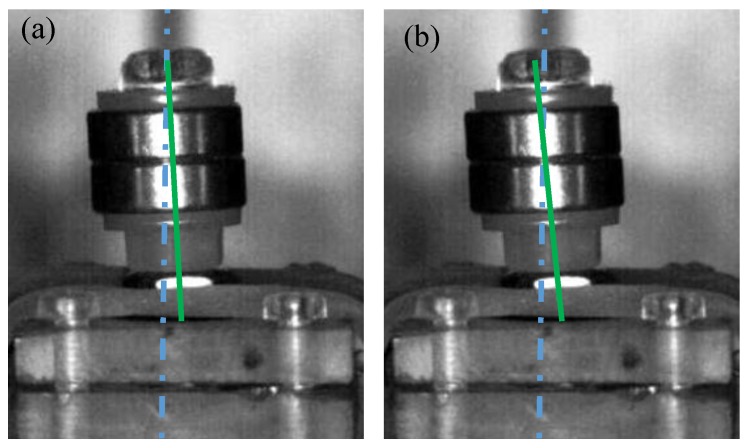
The captured images (front view) show the beam motion of the AC magnetic sensor under: (**a**) zero magnetic field (initial state) and (**b**) *x*-axis magnetic field. Note: The shooting speed and sensitivity for image capture of the high-speed camera is 500 fps and ISO-1600, respectively. Additionally, for videos, please see the Supplementary Materials for a clearer motion of the sensor under an in-plane magnetic field.

For design validation, the images captured and the movie recorded by, using a high-speed camera, are shown in Figure 5. In Figure 5, by comparing the axial line of the NdFeB magnet (i.e., green line) and the reference line fixed on the background (i.e., blue line), we observe that the NdFeB magnet of the sensor experiences a small angle rotation with a small horizontal displacement. This shows that the *x*-axis and *y*-axis piezoelectric beams of the sensor experience slight bending and torsion, respectively (i.e., the sensor design is proven).

**Figure 6:**
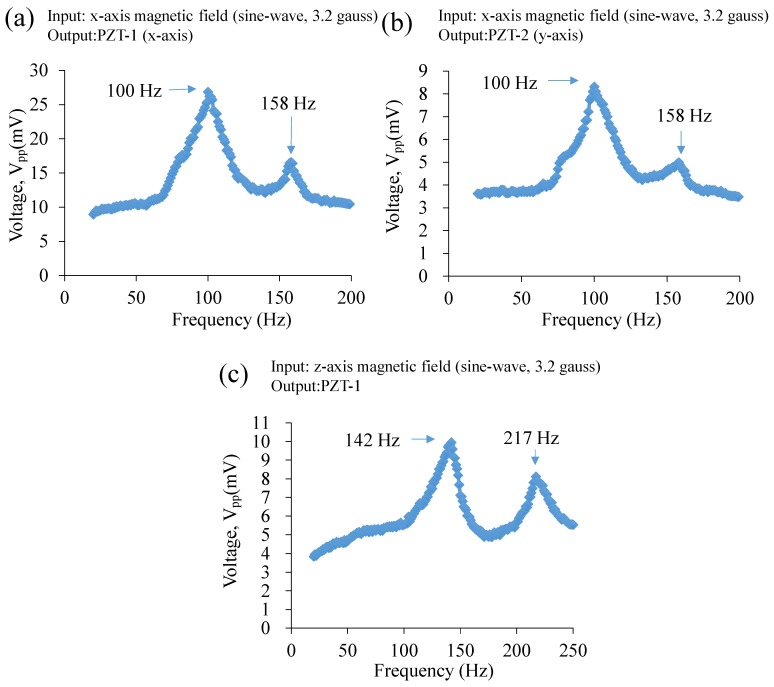
Experimental results of the measurement of the magnetic-field-induced resonant frequency: The voltage output vs. magnetic-field-frequency plots of the AC magnetic sensor under: (**a**,**b**) *x*-axis magnetic field (sine-wave, 3.2 gauss), and (**c**) *z*-axis magnetic field (sine-wave, 3.2 gauss).

Regarding to the resonant frequency measurement, we first measure the magnetic-field-induced resonant frequency. That is, we apply the magnetic field with same field magnitude but varying the magnetic field’s frequency (with an incremental step of 1 Hz) from 20 Hz to 200 Hz for the *x*-axis magnetic field test and from 20 Hz to 250 Hz for the *z*-axis magnetic field test, respectively. The measured voltage outputs of the sensor under the magnetic field with different frequencies are plotted in Figure 6. Through the plots, the magnetic-field-induced resonant frequency of the sensor is obtained when the voltage output is maximized. The results show the resonant frequency of the sensor is 100 Hz and 142 Hz for the *x*-axis and *z*-axis magnetic field test, respectively.

**Figure 7:**
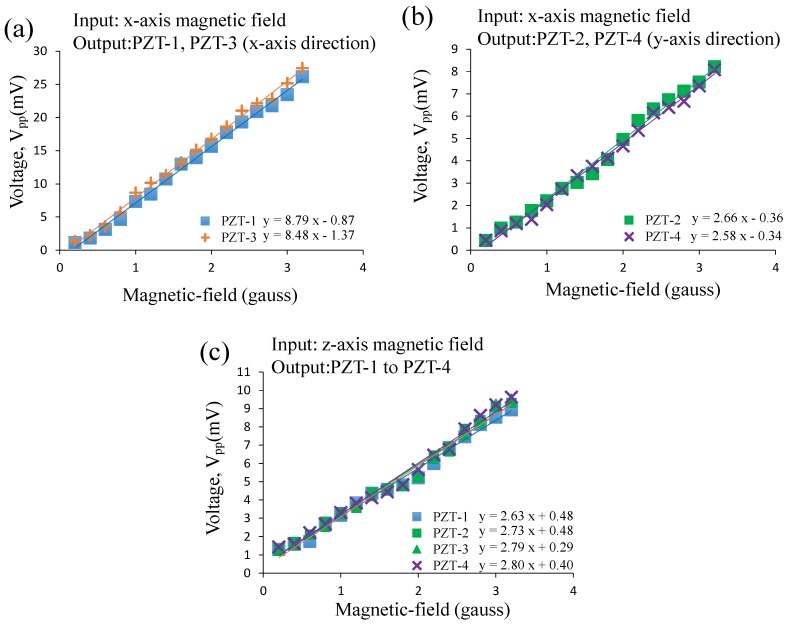
Experimental results of the performance test of the AC magnetic sensor. The voltage outputs of the AC magnetic sensor under: (**a**,**b**) the *x*-axis magnetic fields (sine-wave, 100 Hz, 0.2–3.2 gauss), and (**c**) the *z*-axis magnetic fields (sine-wave, 142 Hz, 0.2–3.2 gauss).

After the resonant frequencies of the sensor were obtained, and we applied the *x*-axis and *z*-axis magnetic fields with different magnitudes at the sensor’s resonant frequency to the sensor, respectively. The voltage outputs versus magnetic-field magnitudes are plotted and correlated by curve fitting, as shown in Figure 7. In Figure 7, the voltage outputs have a good linearity with the magnetic-field magnitudes. The voltage outputs with the sensitivities of the PZT-1, PZT-2, PZT-3, and PZT-4 of the sensor under the *x*-axis magnetic field (sine-wave, 100 Hz, 0.2–3.2 gauss) are 1.13–26.15 mV with 8.79 mV/gauss, 0.43–8.21 mV with 2.66 mV/gauss, 1.33–27.43 mV with 8.48 mV/gauss, and 0.45–8.08 mV with 2.58 mV/gauss, respectively. The voltage outputs of the PZT sheets along the *x*-axis (PZT-1, PZT-3) are approximately three-times larger than those of the PZT sheets along the *y*-axis (PZT-2, PZT-4). This is reasonable, as researchers have reported that the piezoelectric output of PZT sheets in the bending mode is larger than the torsion mode [31], and our sensor’s PZT sheets do experience this. Thus, the voltage outputs of the PZT sheets along the *x*-axis (PZT-1, PZT-3) and the *y*-axis (PZT-2, PZT-4) can be correlated to indicate the magnitude of the applied *x*-axis and *y*-axis magnetic field, respectively. In Figure 7c, the voltage outputs with the sensitivities of PZT-1, PZT-2, PZT-3, and PZT-4 of the sensor under the *z*-axis magnetic field (sine-wave, 142 Hz, 0.2–3.2 gauss) were 1.31–8.92 mV with 2.63 mV/gauss, 1.33–9.31 mV with 2.73 mV/gauss, 1.22–9.55 mV with 2.79 mV/gauss, and 1.44–9.63 mV with 2.80 mV/gauss, respectively. Because of the geometric symmetry of the cross-linked beams, the voltage output of each PZT sheet is similar. Therefore, the voltage outputs of any of the four PZT sheets can be correlated in order to indicate the magnitude of the applied *z*-axis magnetic field. Finally, according to these results, our sensor successfully achieves three-axis magnetic field sensing. Furthermore, because the voltage outputs are large and are directly produced by the PZT sheets under different magnetic fields, the sensor is able to perform the energy harvesting function.

The sensitivity of our magnetic-force-interaction-based AC magnetic sensor, and other representative magnetic-force-interaction-based magnetic sensors, are summarized in Table 2 for comparison. Because the volume of each sensor is different and the voltage output is proportional to the volume of the sensor/sensing-element (due to the definition of power density), the sensitivity of each sensor has to be normalized for a reasonable comparison. For normalization, the sensitivity of the sensor is normalized using the volume of the movable structure and the entire device. As shown in Table 2, the normalized sensitivity of our sensor, and those of the representative sensors, are in a comparable level (note: for the noise and temperature issues, please see Supplemental Materials). Moreover, our sensor is the only one that uses a compact single mechanism in order to achieve three-axis magnetic sensing, while the other sensors have to use three, individual, single-axis magnetic sensors in order to achieve the same purpose. This demonstrates that our sensor has more advantages (i.e., compact single-mechanical mechanism, dual functions, less volume, easier assembly works, etc.) than the other sensors.

### 5.2. Piezoelectric/Vibrational Energy-Harvesting Functions

**Figure 8:**
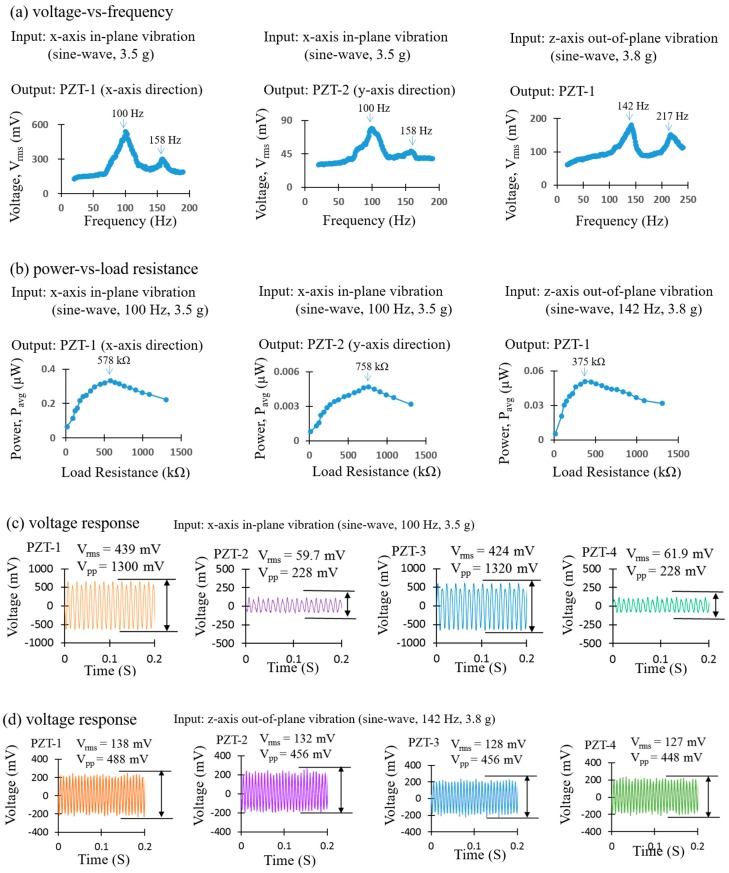
Test results of the piezoelectric/vibrational energy-harvesting function of the AC magnetic sensor: (**a**) voltage vs. frequency plots and (**b**) power vs. load resistance plots. The voltage outputs of each piezoelectric lead-zirconate-titanate (PZT) sheet of the sensor under (**c**) *x*-axis vibration and (**d**) *z*-axis vibration.

Through vibration-induced resonant frequency testing (i.e., varying the applied vibration’s frequency, from 20 Hz to 190 Hz, for *x*-axis vibrations, and from 20 Hz to 240 Hz for *z*-axis vibrations, and then recording the voltage responses), the vibration-induced resonant frequency of the sensor is 100 Hz for the *x*-axis vibration and 142 Hz for the *z*-axis vibration, as shown in Figure 8a. When comparing Figure 8a and Figure 6, a slight asymmetry is shown to exist between the curves; this is caused by using different excitations (i.e., magnetic-fields or vibrations) [32]. Furthermore, through optimum load testing [27], the optimum load resistance of PZT-1 and PZT-2 of the sensor, under the *x*-axis vibration, is 578 kΩ and 758 kΩ, respectively, while the optimum load resistance of each PZT sheet of the sensor under the *z*-axis vibration is 375 kΩ, as shown in Figure 8b. Furthermore, in Figure 8c, the root-mean-square (RMS) voltage outputs of PZT-1, PZT-2, PZT-3, and PZT-4 of the sensor under *x*-axis vibration (sine-wave, 100 Hz, 3.5 g, tested at resonant and optimum loads), are approximately 439 mV, 59.7 mV, 424 mV, and 61.9 mV, respectively. In Figure 8d, the RMS voltage outputs of PZT-1, PZT-2, PZT-3, and PZT-4 of the sensor under *z*-axis vibration (sine-wave, 142 Hz, 3.8 g, tested at resonant and optimum loads) are approximately 138 mV, 132 mV, 128 mV, and 127 mV, respectively. By using the equation P = V^2^/R [27,33] (P: Average power output, V: RMS voltage output, R: Load resistance), the average power outputs of PZT-1, PZT-2, PZT-3, and PZT-4 of the sensor under the *x*-axis vibration (sine-wave, 100 Hz, 3.5 g, tested at resonant and optimum loads) are approximately 0.333 μW, 0.005 μW, 0.311 μW, and 0.005 μW, respectively. The average power outputs of PZT-1, PZT-2, PZT-3, and PZT-4 of the sensor under the *z*-axis vibration (sine-wave, 142 Hz, 3.8 g, tested at resonant and optimum loads) are approximately 0.051 μW, 0.046 μW, 0.044 μW, and 0.043 μW, respectively. According to our previous work [27], there are many sensors that consume ultra-low amounts of power nowadays, in which only few nW (or even a few pW) are sufficient to drive them. The power harnessed by our AC magnetic sensor is able to drive these nW–pW level sensors. These results confirm that our AC magnetic sensor possesses a decent three-axis piezoelectric/vibrational energy-harvesting function.

**Figure 9:**
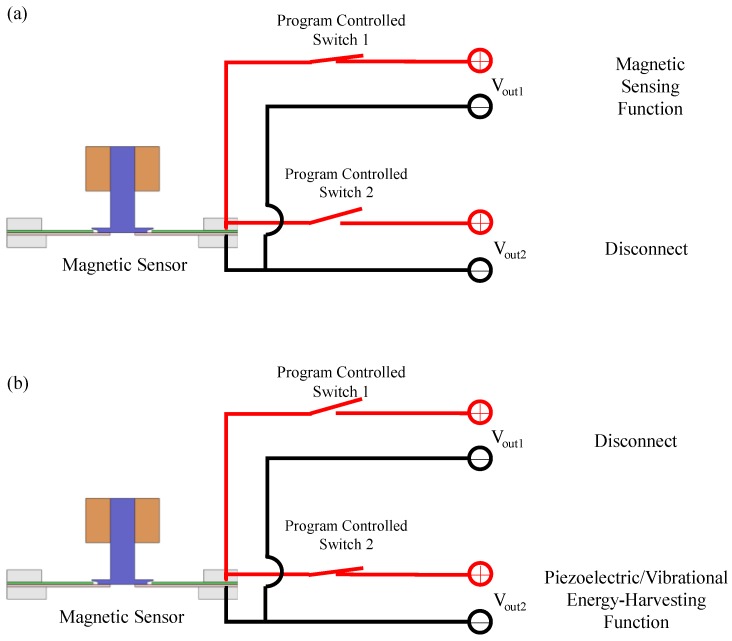
Illustration of the electrical switching approach, which uses a set of program-controlled switches and setting protocols to switch between the functions of the sensor: (**a**) enabling the sensing function while disabling the energy-harvesting function, and (**b**) disabling the sensing function while enabling the energy-harvesting function.

Finally, after the magnetic sensing function and the piezoelectric/vibrational energy-harvesting function of our AC magnetic sensor were performed, we provided guidelines of our sensor for applications. In general, our sensor’s magnetic-sensing function is enabled when using applications that typically require magnetic sensors (for example, vehicle/traffic monitoring, processes monitoring, and security system applications need to use three-axis magnetic sensors). However, the magnetic sensing function can be disabled when the application is turned off. Thus, in this condition, the vibrational energy-harvesting function of the senor can be enabled in order to harness ambient vibrational energy. To switch (i.e., enable or disable) between the magnetic sensing function and the vibration energy-harvesting function, a set of program-controlled switches are used (shown in Figure 9). When turning on the application, program-controlled switch 1 is set to a closed state, and switch 2 is set to an open state, as shown in Figure 9a. Therefore, the sensor enables the magnetic-sensing function and, thus, the voltage output is transmitted to V_out1_. When the magnetic-sensing function is disabled, or is in standby mode, program-controlled switch 1 is set to an open state and switch 2 is set to a closed state, as shown in Figure 9b. Therefore, the sensor enables the piezoelectric/vibrational energy-harvesting function, and, thus, the voltage output is transmitted to V_out2_. By using the setting protocols, the sensor achieves switching between the magnetic-sensing function and the piezoelectric/vibrational energy-harvesting function. The switching significantly improves the energy utilization of the sensor (note: some researchers may have a concern on a mechanical-coupling/decoupling issue when our sensor utilizes a single mechanical-mechanism to demonstrate two functions. For this issue, please see Supplemental Materials).

To illustrate the importance of the two functions (sensing and harvesting) of our sensor, we found some applications that require magnetic field sensing, and that also produce vibrations for energy harvesting; this is to say that these applications match our sensor’s features on both magnetic-field sensing and vibrational energy-harvesting. The first application was intelligent vehicle monitoring [34]. Magnets are fixed to the movable structures of the vehicle’s components. When the vehicle components are used, AC magnetic fields are produced. A magnetic sensor is needed to sense the AC magnetic fields in order to monitor the operating conditions of vehicle components (such as steering-torque sensing/monitoring and motor-position sensing/monitoring). In addition, while operating vehicle components, the moveable structures of the vehicle components generate a periodic rotating movement, which causes rotational vibration. In addition, while driving the vehicle, three axial vibrations are generated in the vehicle. Due to these vibrations, our sensor can detect magnetic fields and also harvest/convert the vibrations into electrical energy. The second application used magnetic sensors for vibration sensing in milling-machining monitoring [35]. Wear/damage to ball bearings from machining spindles can produce a local magnetic flux change. A magnetic sensor is needed in order to sense the magnetic flux change to monitor the health of the ball bearings and the spindle. In addition, while milling, rotational vibration occurred in the spindle and three axial vibrations occurred in the milling-machine. According to this, our sensor can detect magnetic fields, and also harvest/convert vibrations to electrical energy. The third application is a speed measurement of a differential gear system [36]. Rotation of the rotary gear produces periodic and alternative magnetic fluxes. A magnetic sensor is needed in order to sense the periodic/alternative magnetic fluxes, so as to measure the rotational speed of the rotary gear. In addition, while operating the rotary gear, rotational motion is produced by the gears and three axial vibrations are produced by the entire gears system. Accordingly, our sensor can detect the magnetic fields and also harvest/convert the motion and vibrations into electrical energy. In summary, our sensor’s features can match the above-mentioned applications; which is to say that our sensor can detect AC magnetic fields produced by the critical components of the vehicle/machine/system, and also harvest the produced vibrations. In addition, when the magnetic-sensing and energy-harvesting functions are performed by the sensor, some researchers may have a concern on the sensors’ power-budget analysis (i.e., the comparison between energy required by the sensor read-out electronics and the energy that can be potentially harvested by the sensor). For the power-budget analysis of our sensor, please see Supplemental Materials.

## 6. Conclusions

In this paper, a miniature magnetic-force-based, three-axis AC magnetic sensor with piezoelectric/vibrational energy-harvesting functions was successfully demonstrated. By using a high-speed camera, the motion of the movable structure of the magnetic sensor was qualitatively confirmed, and, thus, our design was verified. Furthermore, the results of the quantitative tests showed that the sensor successfully achieved three-axis magnetic-field sensing and three-axis piezoelectric/vibrational energy harvesting. According to these features, the sensor could be an important and alternative approach for three-axis AC magnetic sensors possessing energy harvesting functions for intelligent vehicle/traffic monitoring, processes monitoring, security systems, and so on.

## Figures and Tables

**Table 1:** The dimensions of the AC magnetic sensor and its components.

The Sensor and Components	Size	Weight
Sensor	(length × width × thickness)	7.1 g
	2 × 2 × 2 cm^3^	
CuBe Sheet	(length × width × thickness)	0.01 g
(sandwiched by mechanical clamp)	3.5 × 3.5 × 0.25 mm^3^	
PZT Sheet	(length × width × thickness)	0.06 g
(sandwiched by mechanical clamp)	3.5 × 3.5 × 1 mm^3^	
CuBe Sheet	(length × width × thickness)	0.02 g
(outside of mechanical clamp)	5.5 × 3.5 × 0.25 mm^3^	
PZT Sheet	(length × width × thickness)	0.07 g
(outside of mechanical clamp)	4 × 3.5 × 1 mm^3^	
Magnet	(radius^2^ × π × high)	2.27 g
	4.35^2^ × π × 6 mm^3^	
	With a 1^2^ × π × 6 mm^3^ hole	
Connector	(radius^2^ × π × high)	0.01 g
	4.0^2^ × π × 0.49 mm^3^	

**Table 2:** Comparison of the sensitivity of our, and other representative, magnetic-force-interaction-based magnetic sensors.

Magnetic Sensors	Our Sensor	Han et al. [21]	Yu et al. [22]
Features	Three-Axis	Single-Axis	Single-Axis
Vol_MS_ (mm^3^)	103.27	2460 ^a^	1733.33 ^a^
Vol_Sensor_ (mm^3^)	8000	25,800 ^a^	36,000 ^a^
S_in-plane_ (mV/gauss)	PZT-1: 8.79 PZT-3: 8.48	Sample S1 ^b^: 790 ^a^ Sample S2 ^b^: 920 ^a^ Sample S3 ^b^: 960 ^a^	N/A
Normalized S_in-plane_ by Vol_MS_^c^	PZT-1: 1.02 ^e^ PZT-3: 0.98 ^e^	Sample S1 ^b^: 3.84 ^a,e^ Sample S2 ^b^: 4.48 ^a,e^ Sample S3 ^b^: 4.66 ^a,e^	N/A
Normalized S_in-plane_ by Vol_Sensor_^d^	PZT-1: 1.02 ^e^ PZT-3: 0.98 ^e^	Sample S1 ^b^: 9.46 ^a,e^ Sample S2 ^b^: 11.01 ^a,e^ Sample S3 ^b^: 11.49 ^a,e^	N/A
S_out-of-plane_ (mV/gauss)	PZT-1: 2.63 PZT-2: 2.73 PZT-3: 2.79 PZT-4: 2.80	N/A	7 ^a^
Normalized S_out-of-plane_ by Vol_MS_^c^	PZT-1: 0.96 ^f^ PZT-2: 0.99 ^f^ PZT-3: 1.01 ^f^ PZT-4: 1.02 ^f^	N/A	0.15 ^a,f^
Normalized S_out-of-plane_ by Vol_Sensor_^d^	PZT-1: 0.96 ^f^ PZT-2: 0.99 ^f^ PZT-3: 1.01 ^f^ PZT-4: 1.02 ^f^	N/A	0.19 ^a,f^

^a^ Value estimated or extrapolated from data in reference; ^b^ Sample S1, S2, and S3 uses same pair of PZT cantilever beams but with different gap/spacing distance of 4 mm, 8 mm, and 12 mm, respectively; ^c^ Divided by the volume of the moveable structure; ^d^ Divided by the volume of the sensor. The single-axis sensor has to combine with two single-axis sensors to achieve 3-axis magnetic field sensing (i.e., thus the volume is increased 3-times); ^e^ Normalized by setting the average sensitivity of two PZTs of our sensor (i.e., average: 8.635 mV/gauss) as 1 for the normalization base; ^f^ Normalized by setting the average sensitivity of four PZTs of our sensor (i.e., average: 2.738 mV/gauss) as 1 for the normalization base. Vol_MS_: volume of the movable structure of the sensor; Vol_Sensor_: volume of the sensor; S_in-plane_: In-plane sensitivity of the sensor tested in *x*-axis magnetic field; S_out-of-plane_: out-of-plane sensitivity of the sensor tested in *z*-axis magnetic field.

## Supplementary Materials

The Supplementary Materials are available online at http://www.mdpi.com/1424-8220/17/2/308/s1.
