# Supplementary material for: A Miniature Magnetic-Force-Based Three-Axis AC Magnetic Sensor with Piezoelectric/Vibrational Energy-Harvesting Functions
Source: Sensors (Basel). 2017 Feb 8;17(2):308. doi: 10.3390/s17020308 (PMC5336095; doi:10.3390/s17020308)
Supplement: Supplementary file 1 [file sensors-17-00308-s001.zip › sensors-17-00308-suppl.pdf]

Supplementary Materials: A Miniature Magnetic-Force-Based Three-Axis AC Magnetic Sensor with Piezoelectric/Vibrational Energy-Harvesting Functions

Chiao-Fang Hung, Po-Chen Yeh and Tien-Kan Chung

Supplemental Materials No.1

Two Specific Applications for 3-Axis Magnetic Fields

Among many industrial applications, we provide two specific applications that can more clearly show the practical applications of our sensor. First, a three-axis current sensor for evaluating the integrity of concentric neutrals in underground power distribution cables [1]: The application proposes the use of the three-axis anisotropic magnetoresistive (AMR) sensor in order to sense the three-axis magnetic field of underground power distribution cables. From the measured three-axis magnetic-field information, the individual concentric neutral can be accurately determined in order to predict whether it is intact or broken. Therefore, this current-sensing system is a very suitable application for our sensor. Second, a motion-tracking application using an AC magnetic field sensing system [2]: In the AC magnetic-sensing system, a reference transducer (not moving) emits the reference AC magnetic field, and a sensing transducer (moving) receives the AC magnetic field. By analyzing the three-axis AC magnetic field signals received by the sensing transducer, the relative position between the sensing transducer and the reference transducer can be obtained. Both transducers are composed of three orthogonal coils that emit or receive AC magnetic fields. The sensing transducer has the same function as our three-axis AC magnetic sensor; thus, this motion-tracking system is also a very suitable application for our sensor. Finally, according to developing trend of smart sensing technology, the above-mentioned applications can be further integrated into a wireless transmission module in order to become a wireless sensor module. However, the wireless sensor module is sometimes located in hard-to-reach areas [3] and, thus, has power supply problems [4]. To solve this problem, both the self-powering and the energy-harvesting functions of our magnetic sensor are good solutions. Our sensor's features (i.e., self-powered, three-axis, AC, and magnetic field sensing) exactly match the needs of the above-mentioned wireless sensing applications. Hence, the above-mentioned applications are also very suitable for our sensor.

1. Seidel, M.; Paprotny, I.; Krishnan, K.; White, R.; Evans, J. AMR Current Sensors for Evaluating the Integrity of Concentric Neutrals in In-Service Underground Power Distribution Cables. In Proceedings of the 2010 IEEE International Symposium on Electrical Insulation (ISEI), San Diego, CA, USA, 6–9 June 2010.
2. Sosnicki, O.; Porchez, T.; Michaud, G.; Bencheikh, N.; Claeyssen, F. AC magnetic field detection system applied to motion tracking. In Proceedings of the Sensoren und Messsysteme 2010-15. ITG/GMA-Fachtagung, Nürnberg, Germany, 18–19 May 2010.
3. Regini, E.; Rosing, T.S. An Energy Efficient Wireless Communication Mechanism for Sensor Node Cluster Heads. In Proceedings of the 2009 International Conference on Intelligent Sensors, Sensor Networks and Information Processing (ISSNIP 2009), Melbourne, Australia, 7–10 December 2009.
4. Chen, W.; Cao, Y.L.; Xie, J. Piezoelectric and electromagnetic hybrid energy harvester for powering wireless sensor nodes in smart grid. *J. Mech. Sci. Technol.* **2015**, *29*, 4313–4318.

Supplemental Materials No. 2

Noise and Temperature Analyses

We have to mention that there is no comparison of noise and temperature analyses in Table 2, because the analyses were not necessary for the magnetic-force-based magnetic sensors. The reason for this is described below. We conducted a literature review regarding noise and temperature analyses of magnetic-force-based magnetic sensors (as our sensor falls in this category). First of all, we found that some magnetoelectric magnetic sensors [5,6] consist of a similar piezoelectric/magnetic-material configuration as our sensor. These sensors use the direct magnetoelectric effect to convert applied magnetic fields to mechanical strain, and, eventually, to piezoelectric voltage outputs. Because these sensors are strain-mediated, the voltage outputs of these sensors are very small. Because of this, influences caused by noise and temperature must be analyzed. However, while the configuration of these sensors and our sensor are similar, the principle of these sensors and our sensor are different. Our sensor uses magnetic-force-interaction to convert applied magnetic fields to mechanical beam deflection, and eventually to piezoelectric voltage outputs. Because our sensor is deflection mediated, the voltage outputs of our sensor are large (larger and easier to distinguish than noise). Because of this, influences caused by the noise and temperature do not need to be analyzed. Moreover, we found that there were no noise and temperature analyses in the literature of representative magnetic-force-interaction magnetic sensors [7–9] (in which the material configuration and operation principles were the same as those of our sensor). This is because the magnetic-force-based magnetic sensors use movable cantilever beams in order to convert magnetic fields to large mechanical beam deflections, and, consequently, to large piezoelectric voltage outputs (i.e., the same as our sensor). Therefore, we can conclude that the output signals of the magnetic-force-interaction magnetic sensors (including our sensor) are less likely to be influenced by noise and temperature. Thus, we did not analyze noise and temperature issues; this information is not provided in Table 2.

5. Marauska, S.; Jahns, R.; Kirchhof, C.; Claus, M.; Quandt, E.; Knochel, R.; Wagner, B. Highly sensitive wafer-level packaged MEMS magnetic field sensor based on magnetoelectric composites. *Sens. Actuators A-Phys.* **2013**, *189*, 321–327.
6. Jahns, R.; Greve, H.; Woltermann, E.; Quandt, E.; Knochel, R. H. Noise performance of magnetometers with resonant thin-film magnetoelectric sensors. *IEEE Trans. Instrum. Meas.* **2011**, *60*, 2995–3001.
7. Cui, N.Y.; Wu, W.W.; Zhao, Y.; Bai; S.; Meng, L.X.; Qin, Y.; Wang, Z.L. Magnetic Force Driven Nanogenerators as a Noncontact Energy Harvester and Sensor. *Nano Lett.* **2012**, *12*, 3701–3705.
8. Han, J.C.; Hu, J.; Wang, Z.X.; Wang, S.X.; He, J.L. Magnetoelectric effect in shear-mode Pb(Zr, Ti)O_3/NdFeB composite cantilever. *Appl. Phys. Lett.* **2015**, *106*, 182901.
9. Yu, A.F.; Song, M.; Zhang, Y.; Kou, J.Z.; Zhai, J.Y.; Wang, Z.L. A self-powered AC magnetic sensor based on piezoelectric nanogenerator. *Nanotechnology* **2014**, *25*, 455503.

Supplemental Materials No. 3

Mechanical-Decouple Issue

We noticed that the two functions (sensing and harvesting) of our sensor cannot be mechanically decoupled, as both functions rely on one magnetic/mechanical moveable structure (i.e., the magnet being physically attached to the cantilevers). This cannot avoid interference between the applied magnetic field and the vibrations. Moreover, researchers recently reported that some magnetic-force-interaction-based magnetic sensors possess dual-functions (i.e., having magnetic sensing and energy harvesting functions) [10–19]. These sensors use a single mechanical-mechanism for both magnetic sensing and energy harvesting functions, as does our sensor; however, in these articles of below sensors, researchers also did not investigate (and also did not have any comment on) the interference between the magnetic field and the vibrations. Thus, we believe that these sensors are in the same condition as ours. That is, the sensors cannot avoid interference between the magnetic field and the vibrations, and, consequently, the interference cannot be mechanically decoupled. Nevertheless, these articles still claim that their sensors have both magnetic sensing and energy harvesting functions. Because of this, we propose a "non-mechanical" switching approach (such as an electrical switching approach) for users to switch between both mechanical-coupled functions. Finally, in contrast to our approach, some researchers proposed another practical approach in which they physically/mechanically separate each function of each individual device in one sensing system (i.e., the sensing system uses one individual magnetic sensor for magnetic sensing and one individual energy harvester for energy harvesting in order to achieve both functionalities) [20]. Therefore, the sensing system can have two functions, while decoupling the interference between the two functions. This approach significantly increases the device volume and also requires a great deal of effort for additional integration/assembly/packaging processes for system integration. That is to say that the disadvantage of this approach is the advantage of our approach. Although our approach has interference issues (it cannot be mechanically decoupled), our approach has many advantages from system integration/assembly/packaging perspectives. This indicates that our approach is an important alternative for developing sensors that have magnetic sensing and vibration energy harvesting functions.

10. Cui, N.Y.; Wu, W.W.; Zhao, Y.; Bai, S.; Meng, L.X.; Qin, Y.; Wang, Z.L. Magnetic Force Driven Nanogenerators as a Noncontact Energy Harvester and Sensor. *Nano Lett.* **2012**, *12*, 3701–3705.
11. Han, J.C.; Hu, J.; Wang, Z.X.; Wang, S.X.; He, J.L. Magnetoelectric effect in shear-mode Pb(Zr, Ti)O_3/NdFeB composite cantilever. *Appl. Phys. Lett.* **2015**, *106*, 182901.
12. Yu, A.F.; Song, M.; Zhang, Y.; Kou, J.Z.; Zhai, J.Y.; Wang Z.L. A self-powered AC magnetic sensor based on piezoelectric nanogenerator. *Nanotechnology* **2014**, *25*, 455503.
13. Zhou, Y.; Priya, S. Near-flat self-biased magnetoelectric response in geometry gradient composite. *J. Appl. Phys.* **2015**, *115*, 104107.
14. Huang, D.Y.; Lu, C.J.; Bing, H. Multipeak self-biased magnetoelectric coupling characteristics in four-phase Metglas/Terfenol-D/Be-bronze/PMN-PT structure. *AIP Adv.* **2015**, *5*, 047140.
15. Yang, C.; Li, P.; Wen, Y.M.; Lu, C.J.; Yang, A.C.; Wang, D.C.; Zhang, J.T.; Zhang, F. Enhanced Multipeak Magnetoelectric Effects in Ferromagnetic/Ferroelectric Composite With H-Shaped Elastic Substrate. *IEEE Trans. Magn.* **2014**, *50*, 4006704.
16. Lu, C.J.; Li, P.; Wen, Y.M.; Yang, A.C.; He, W.; Zhang, J.T.; Yang, J.; Wen, J.; Zhu, Y.; Yu, M. Investigation of magnetostrictive/piezoelectric multilayer composite with a giant zero-biased magnetoelectric effect. *Appl. Phys. A-Mater. Sci. Process.* **2013**, *113*, 413–421.
17. Huang, D.Y.; Lu, C.J.; Bing, H. Self-biased magnetoelectric coupling characteristics of three-phase composite transducers with nanocrystallin soft magnetic alloy. *Appl. Phys. A-Mater. Sci. Process.* **2015**, *120*, 115–120.
18. Han, J.C.; Hu, J.; Wang, S.X.; He, J.L. A novel cylindrical torsional magnetoelectric composite based on d(15) shear-mode response. *J. Phys. D-Appl. Phys.* **2015**, *48*, 045001.

19. Grechishkin, R.M.; Kaplunov, I.A.; Ilyashenko, S.E.; Lebedev, G.A.; Malyshkina, O.V.; Mamkina, N.O.; Zalyotov, A.B. Magnetoelectric Effect in Metglas/Piezoelectric Macrofiber Composites. *Ferroelectrics* **2011**, *424*, 78–85.
20. Paprotny, I.; Leland, E.; Sherman, C.; White, R.M.; Wright, P.K. Self-powered MEMS sensor module for measuring electrical quantities in residential, commercial, distribution and transmission power systems. In Proceedings of the 2010 IEEE Energy Conversion Congress and Exposition (ECCE), Atlanta, GA, USA, 12–16 September 2010.

Supplemental Materials No. 4

Power Budget Analysis

In our testing setup, we used a lock-in amplifier to distinguish voltage signals from electromagnetic induction noise. However, using lock-in amplifier consumes lots of energy and thus the result of the power budget analysis will be worse. Therefore, we conduct the power budget analysis of our sensor by estimating lock-in amplifier's power consumption. The analysis method is based on the power budget section of our previous published article [21]. The details of the power budget analysis are described in following paragraphs.

Due to the similar configuration of our sensor and our previous work [22], the power output of the energy-harvesting elements of our sensor should be estimated by using the connect-in-series approach [22]. When the PZT sheets are connected in series, the corresponding optimum load resistance is changed accordingly. Therefore, the power output of the connect-in-series PZT sheets is not directly the sum of the power output of each PZT sheet, but is a proportional relation to the sum of the power output of each PZT sheet. According to our previous work, when the two connect-in-series PZT sheets under in-plane vibration (Sine-wave, 100 Hz, 3.5 g), the optimum load resistance rise from 578 kΩ to 834 kΩ, and the connect-in-series voltage output is 0.86 times of the sum of each voltage output. Based on the proportional relation, the connect-in-series voltage output of our sensor is estimated as increased from 439 mV to 742 mV. According to our previous work, when the four connect-in-series PZT sheets under out-of-plane vibration, the optimum load resistance rise from 375 kΩ to 909 kΩ, and the connect-in-series voltage output is 1.23 times of the sum of each voltage output. Based on the proportional relation, the estimated connect-in-series voltage output of our sensor is increased from 138 mV to 646 mV. Furthermore, according to the above estimated connect-in-series voltage outputs and the equation P = V2/R [22], the connect-in-series power output of our sensor is estimated. Through this estimation, the estimated voltage output and power output of two connect-in-series PZT sheets of our sensor under the in-plane vibration (Sine-wave, 100 Hz, 3.5 g) is 742 mV and 0.660 μW. The estimated voltage output and power output of four connect-in-series PZT sheets of our sensor under the out-of-plane vibration (Sine-wave, 140 Hz, 3.8 g) is 646 mV and 0.459 μW.

Regarding to the energy required by the sensor read-out electronics, because we use a standard lock-in amplifier, the energy consumption is not optimal. Thus, to estimate the optimal energy consumption, we compare potential cases using: a standard lock-in amplifier [23], a commercially available lock-in amplification IC [24], and a CMOS low-power lock-in amplifier [25]. Note: the specifications of these lock-in amplifiers match our sensor's requirements (i.e., frequency: 20–250 Hz. dynamic reserve: at least 20 dB). However, the power consumption of both standard and commercially available lock-in amplifiers are much larger than that of our sensor. Thus, our sensor cannot directly power these lock-in amplifiers. The power consumption of the CMOS low-power lock-in amplifier is about 417 μW. However, the power output of our sensor under the in-plane and out-of-plane vibration is 0.660 μW and 0.459 μW, respectively. Thus, we have to use the energy storage approach [26] to accumulate/store the harnessed energy of the energy-harvesting elements of our sensor in order to drive the CMOS lock-in amplifier as the sensor's read-out electronics. The details of the energy storage approach for our sensor are described as follows. First, we switch the program-controlled switches to enable/activate the piezoelectric/vibrational energy-harvesting function, and then accumulate/store energy for about 15 minutes (about 908 seconds). After this, we switch the program-controlled switches to the magnetic sensing function, and then use the

accumulated/stored energy to drive the sensor read-out electronics to read an output signal. However, we know this is a rough estimation of the power budget of our sensor. In practical, a smart power management should be conducted to optimize the power budget, but we are not able to perform the power management for optimization. Thus, we only can provide the rough power budget analysis of our sensor (as mentioned above), which is shown in Table S1. In the future, we will use shielded cables for experiments to enable the use of the oscilloscopes (instead of the lock-in amplifier). This will significantly reduce the power consumption of the sensor read-out electronics.

Table S1. The power budget analysis of our sensor.

**Energy Harvesting**				
Input Vibration	X-axis (in-plane) vibration: Sine-wave, 100 Hz, 3.5 g		Z-axis (out-of-plane) vibration: Sine-wave, 140 Hz, 3.8 g	
Output	Single PZT	Two PZTs connect-in-series	Single PZT	Four PZTs connect-in-series
R_o (kΩ)	578	834 *	375	909 *
V_{rms} (mV)	439	742 *	138	646 *
P_{avg} (μW)	0.333	0.660 *	0.051	0.459 *

**Power Consumption of Sensor Read-Out Electronics**			
Lock-in Amplifier	Stanford Research Systems, SR830 [23]	Analog Devices, AD630 [24]	CMOS Low-Power Lock-In Amplifier [25]
Frequency	1 mHz to 102 kHz	Up to 1kHz	Up to 128 kHz
Dynamic Reserve	≥100 dB	110 dB	24.7–42 dB
Power	40 W	20 mW	417 μW

Note: R_o: the optimum load resistance; V_{rms}: root-mean-square voltage output; P_{avg}: average power output; * Value estimated from data of our sensor.

21. Chung, T.K.; Yeh, P.C.; Lee, H.; Lin, C.M.; Tseng, C.Y.; Lo, W.T.; Wang, C.M.; Wang, W.C.; Tu, C.J.; Tasi, P.Y.; Chang, J.W. An Attachable Electromagnetic Energy Harvester Driven Wireless Sensing System Demonstrating Milling-Processes and Cutter-Wear/Breakage-Condition Monitoring. *Sensors* **2016**, *16*, 269.
22. Hung, C.F.; Chung, T.K.; Yeh, P.C.; Chen, C.C.; Wang, C.M.; Lin, S.H. A Miniature Mechanical-Piezoelectric-Configured Three-Axis Vibrational Energy Harvester. *IEEE Sens. J.* **2015**, *15*, 5601–5615.
23. MODEL SR830 DSP Lock-In Amplifier. Available online: http://www.thinksrs.com/downloads/PDFs/Manuals/SR830m.pdf (accessed on 24 January 2017).
24. AD630 Balanced Modulator/Demodulator. Available online: http://www.analog.com/media/en/technical-documentation/data-sheets/AD630.pdf (accessed on 24 January 2017).
25. Maya-Hernández, P.M.; Alvarez-Simon, L.C.; Sanz-Pascual, M.T.; Calvo-Lopez, B. An Integrated Low-Power Lock-In Amplifier and Its Application to Gas Detection. *Sensors* **2014**, *14*, 15880–15899.
26. Leland, E.S.; Lai, E.M.; Wright, P.K. A Self-Powered Wireless Sensor for Indoor Environmental Monitoring. In Proceedings of the Wireless Networking Symposium (WNCG), Austin, TX, USA, 20–22 October 2004.
